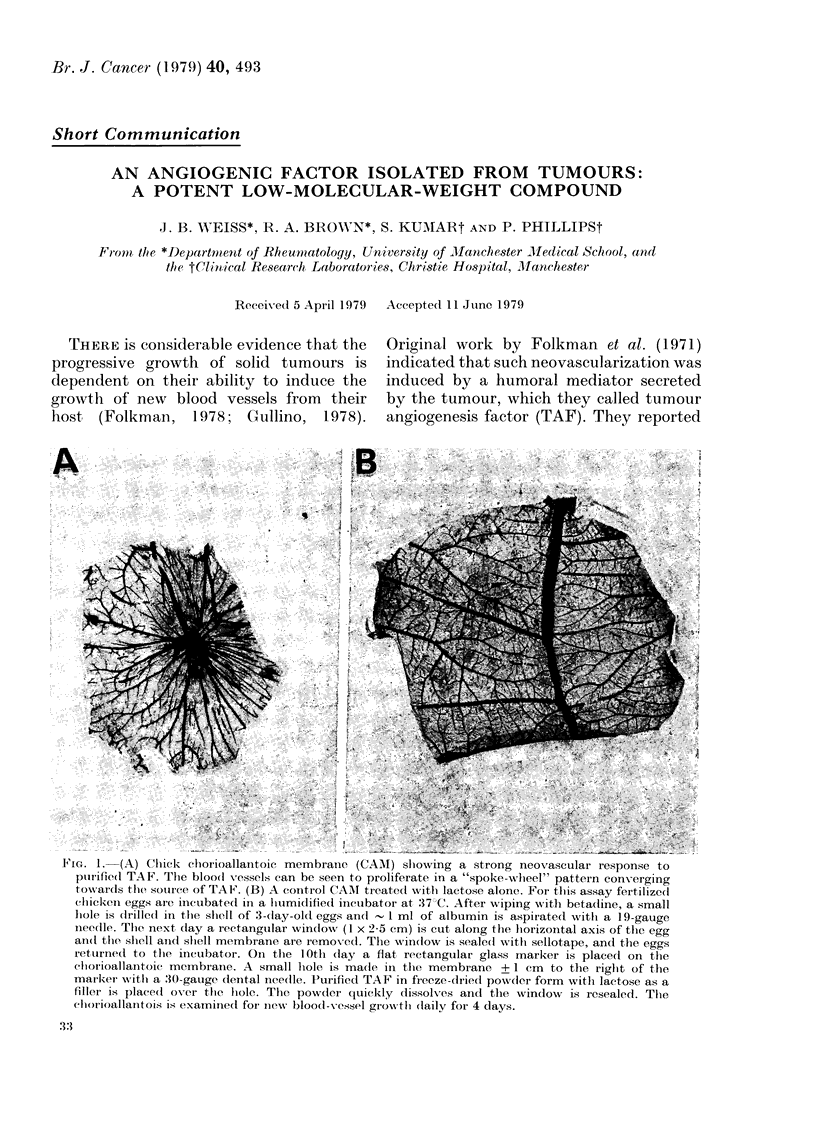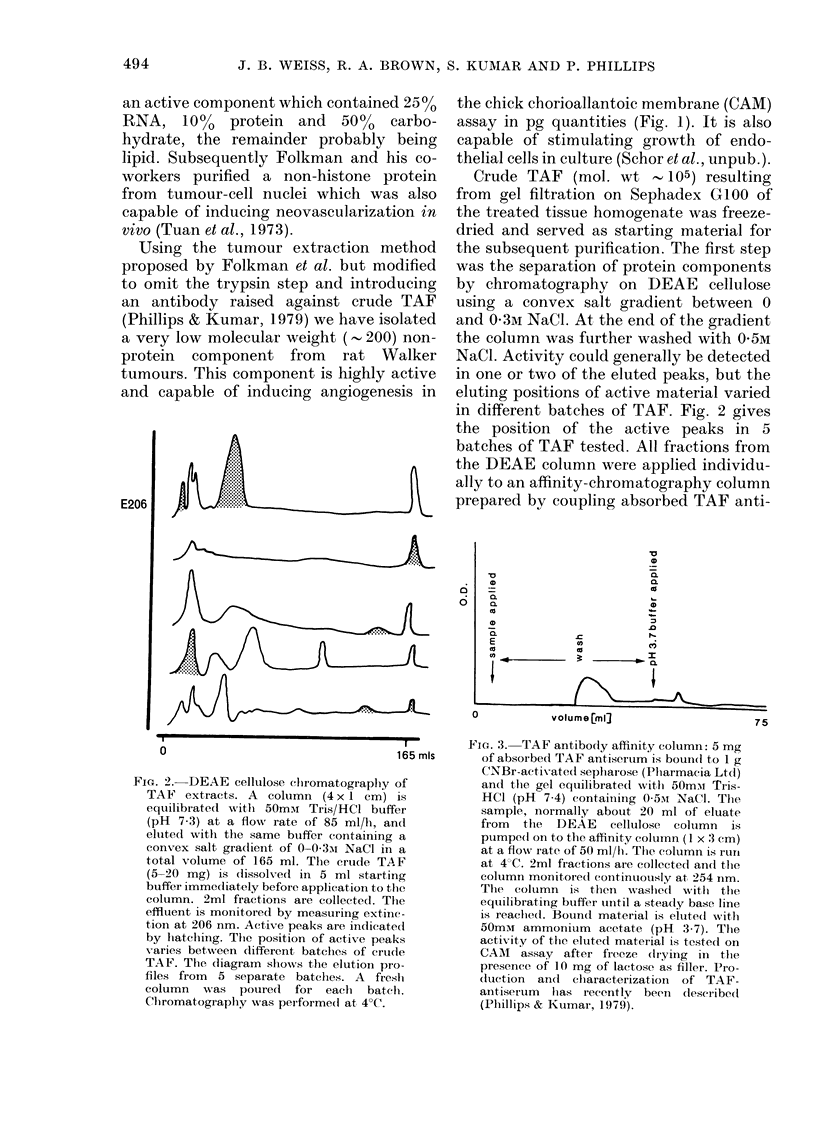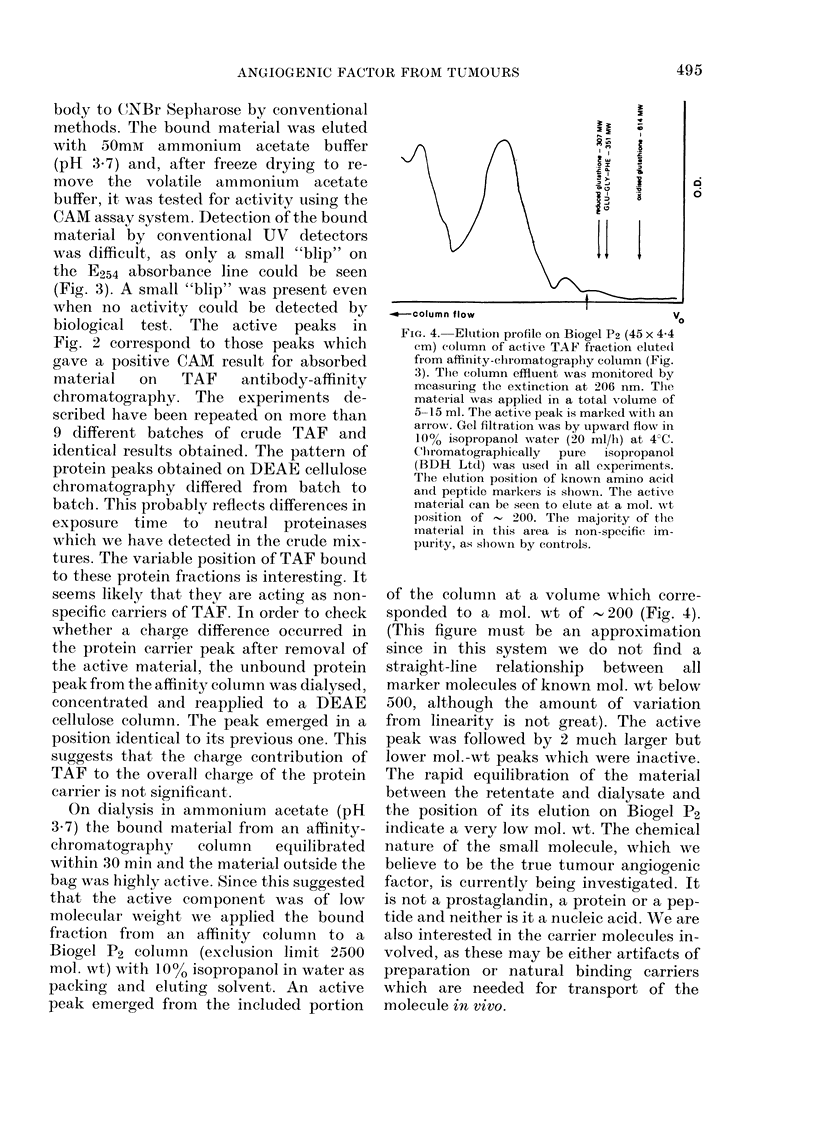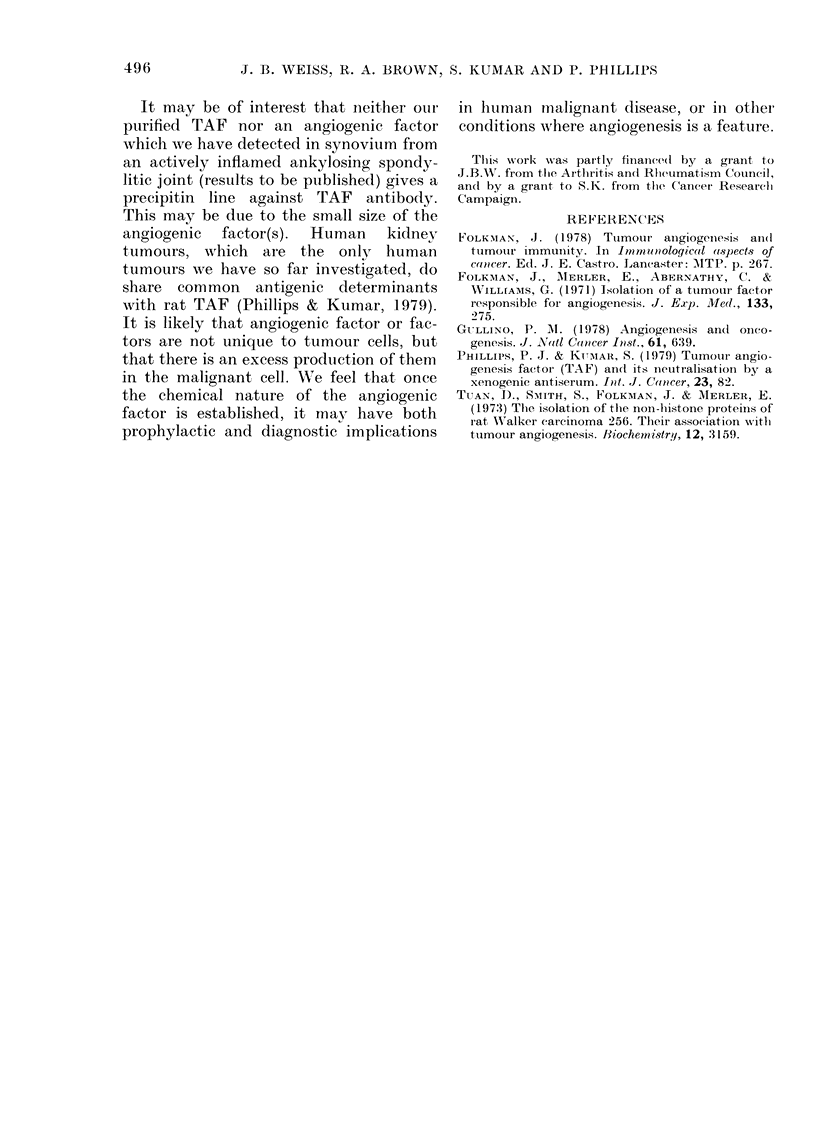# An angiogenic factor isolated from tumours: a potent low-molecular-weight compound.

**DOI:** 10.1038/bjc.1979.206

**Published:** 1979-09

**Authors:** J. B. Weiss, R. A. Brown, S. Kumar, P. Phillips

## Abstract

**Images:**


*Br. J. Cancer* (1979) **40**, 493

***Short Communication***

### AN ANGIOGENIC FACTOR ISOLATED FROM TUMOURS: A POTENT LOW-MOLECULAR-WEIGHT COMPOUND

J. B. WEISS*, R. A. BROWN*, S. KUMAR† AND P. PHILLIPS†

*From the *Department of Rheumatology, University of Manchester Medical School, and the †Clinical Research Laboratories, Christie Hospital, Manchester*

Received 5 April 1979 Accepted 11 June 1979

THERE is considerable evidence that the progressive growth of solid tumours is dependent on their ability to induce the growth of new blood vessels from their host (Folkman, 1978; Gullino, 1978). Original work by Folkman *et al.* (1971) indicated that such neovascularization was induced by a humoral mediator secreted by the tumour, which they called tumour angiogenesis factor (TAF). They reported

FIG. 1.—(A) Chick chorioallantoic membrane (CAM) showing a strong neovascular response to purified TAF. The blood vessels can be seen to proliferate in a "spoke-wheel" pattern converging towards the source of TAF. (B) A control CAM treated with lactose alone. For this assay fertilized chicken eggs are incubated in a humidified incubator at 37°C. After wiping with betadine, a small hole is drilled in the shell of 3-day-old eggs and ~1 ml of albumin is aspirated with a 19-gauge needle. The next day a rectangular window (1 × 2·5 cm) is cut along the horizontal axis of the egg and the shell and shell membrane are removed. The window is sealed with sellotape, and the eggs returned to the incubator. On the 10th day a flat rectangular glass marker is placed on the chorioallantoic membrane. A small hole is made in the membrane ±1 cm to the right of the marker with a 30-gauge dental needle. Purified TAF in freeze-dried powder form with lactose as a filler is placed over the hole. The powder quickly dissolves and the window is resealed. The chorioallantois is examined for new blood-vessel growth daily for 4 days.

an active component which contained 25% RNA, 10% protein and 50% carbohydrate, the remainder probably being lipid. Subsequently Folkman and his co-workers purified a non-histone protein from tumour-cell nuclei which was also capable of inducing neovascularization *in vivo* (Tuan *et al.*, 1973).

Using the tumour extraction method proposed by Folkman *et al.* but modified to omit the trypsin step and introducing an antibody raised against crude TAF (Phillips & Kumar, 1979) we have isolated a very low molecular weight (~200) non-protein component from rat Walker tumours. This component is highly active and capable of inducing angiogenesis in the chick chorioallantoic membrane (CAM) assay in pg quantities (Fig. 1). It is also capable of stimulating growth of endothelial cells in culture (Schor *et al.*, unpub.).

Crude TAF (mol. wt ~$10^5$) resulting from gel filtration on Sephadex G100 of the treated tissue homogenate was freeze-dried and served as starting material for the subsequent purification. The first step was the separation of protein components by chromatography on DEAE cellulose using a convex salt gradient between 0 and 0·3M NaCl. At the end of the gradient the column was further washed with 0·5M NaCl. Activity could generally be detected in one or two of the eluted peaks, but the eluting positions of active material varied in different batches of TAF. Fig. 2 gives the position of the active peaks in 5 batches of TAF tested. All fractions from the DEAE column were applied individually to an affinity-chromatography column prepared by coupling absorbed TAF anti-

FIG. 2.—DEAE cellulose chromatography of TAF extracts. A column (4 × 1 cm) is equilibrated with 50mM Tris/HCl buffer (pH 7·3) at a flow rate of 85 ml/h, and eluted with the same buffer containing a convex salt gradient of 0–0·3M NaCl in a total volume of 165 ml. The crude TAF (5–20 mg) is dissolved in 5 ml starting buffer immediately before application to the column. 2ml fractions are collected. The effluent is monitored by measuring extinction at 206 nm. Active peaks are indicated by hatching. The position of active peaks varies between different batches of crude TAF. The diagram shows the elution profiles from 5 separate batches. A fresh column was poured for each batch. Chromatography was performed at 4°C.

FIG. 3.—TAF antibody affinity column: 5 mg of absorbed TAF antiserum is bound to 1 g CNBr-activated sepharose (Pharmacia Ltd) and the gel equilibrated with 50mM Tris-HCl (pH 7·4) containing 0·5M NaCl. The sample, normally about 20 ml of eluate from the DEAE cellulose column is pumped on to the affinity column (1 × 3 cm) at a flow rate of 50 ml/h. The column is run at 4°C. 2ml fractions are collected and the column monitored continuously at 254 nm. The column is then washed with the equilibrating buffer until a steady base line is reached. Bound material is eluted with 50mM ammonium acetate (pH 3·7). The activity of the eluted material is tested on CAM assay after freeze drying in the presence of 10 mg of lactose as filler. Production and characterization of TAF-antiserum has recently been described (Phillips & Kumar, 1979).

body to CNBr Sepharose by conventional methods. The bound material was eluted with 50mM ammonium acetate buffer (pH 3·7) and, after freeze drying to remove the volatile ammonium acetate buffer, it was tested for activity using the CAM assay system. Detection of the bound material by conventional UV detectors was difficult, as only a small "blip" on the $E_{254}$ absorbance line could be seen (Fig. 3). A small "blip" was present even when no activity could be detected by biological test. The active peaks in Fig. 2 correspond to those peaks which gave a positive CAM result for absorbed material on TAF antibody-affinity chromatography. The experiments described have been repeated on more than 9 different batches of crude TAF and identical results obtained. The pattern of protein peaks obtained on DEAE cellulose chromatography differed from batch to batch. This probably reflects differences in exposure time to neutral proteinases which we have detected in the crude mixtures. The variable position of TAF bound to these protein fractions is interesting. It seems likely that they are acting as non-specific carriers of TAF. In order to check whether a charge difference occurred in the protein carrier peak after removal of the active material, the unbound protein peak from the affinity column was dialysed, concentrated and reapplied to a DEAE cellulose column. The peak emerged in a position identical to its previous one. This suggests that the charge contribution of TAF to the overall charge of the protein carrier is not significant.

On dialysis in ammonium acetate (pH 3·7) the bound material from an affinity-chromatography column equilibrated within 30 min and the material outside the bag was highly active. Since this suggested that the active component was of low molecular weight we applied the bound fraction from an affinity column to a Biogel $P_2$ column (exclusion limit 2500 mol. wt) with 10% isopropanol in water as packing and eluting solvent. An active peak emerged from the included portion

FIG. 4.—Elution profile on Biogel $P_2$ (45 x 4·4 cm) column of active TAF fraction eluted from affinity-chromatography column (Fig. 3). The column effluent was monitored by measuring the extinction at 206 nm. The material was applied in a total volume of 5–15 ml. The active peak is marked with an arrow. Gel filtration was by upward flow in 10% isopropanol water (20 ml/h) at 4°C. Chromatographically pure isopropanol (BDH Ltd) was used in all experiments. The elution position of known amino acid and peptide markers is shown. The active material can be seen to elute at a mol. wt position of ~ 200. The majority of the material in this area is non-specific impurity, as shown by controls.

of the column at a volume which corresponded to a mol. wt of ~200 (Fig. 4). (This figure must be an approximation since in this system we do not find a straight-line relationship between all marker molecules of known mol. wt below 500, although the amount of variation from linearity is not great). The active peak was followed by 2 much larger but lower mol.-wt peaks which were inactive. The rapid equilibration of the material between the retentate and dialysate and the position of its elution on Biogel $P_2$ indicate a very low mol. wt. The chemical nature of the small molecule, which we believe to be the true tumour angiogenic factor, is currently being investigated. It is not a prostaglandin, a protein or a peptide and neither is it a nucleic acid. We are also interested in the carrier molecules involved, as these may be either artifacts of preparation or natural binding carriers which are needed for transport of the molecule *in vivo*.

It may be of interest that neither our purified TAF nor an angiogenic factor which we have detected in synovium from an actively inflamed ankylosing spondylitic joint (results to be published) gives a precipitin line against TAF antibody. This may be due to the small size of the angiogenic factor(s). Human kidney tumours, which are the only human tumours we have so far investigated, do share common antigenic determinants with rat TAF (Phillips & Kumar, 1979). It is likely that angiogenic factor or factors are not unique to tumour cells, but that there is an excess production of them in the malignant cell. We feel that once the chemical nature of the angiogenic factor is established, it may have both prophylactic and diagnostic implications in human malignant disease, or in other conditions where angiogenesis is a feature.

This work was partly financed by a grant to J.B.W. from the Arthritis and Rheumatism Council, and by a grant to S.K. from the Cancer Research Campaign.

### REFERENCES

FOLKMAN, J. (1978) Tumour angiogenesis and tumour immunity. In *Immunological aspects of cancer*. Ed. J. E. Castro. Lancaster: MTP. p. 267.

FOLKMAN, J., MERLER, E., ABERNATHY, C. & WILLIAMS, G. (1971) Isolation of a tumour factor responsible for angiogenesis. *J. Exp. Med.*, **133**, 275.

GULLINO, P. M. (1978) Angiogenesis and oncogenesis. *J. Natl Cancer Inst.*, **61**, 639.

PHILLIPS, P. J. & KUMAR, S. (1979) Tumour angiogenesis factor (TAF) and its neutralisation by a xenogenic antiserum. *Int. J. Cancer*, **23**, 82.

TUAN, D., SMITH, S., FOLKMAN, J. & MERLER, E. (1973) The isolation of the non-histone proteins of rat Walker carcinoma 256. Their association with tumour angiogenesis. *Biochemistry*, **12**, 3159.